# Differential long-term stability of microRNAs and RNU6B snRNA in 12–20 year old archived formalin-fixed paraffin-embedded specimens

**DOI:** 10.1186/s12885-016-3008-4

**Published:** 2017-01-06

**Authors:** Sarah B. Peskoe, John R. Barber, Qizhi Zheng, Alan K. Meeker, Angelo M. De Marzo, Elizabeth A. Platz, Shawn E. Lupold

**Affiliations:** 1 Department of Epidemiology, Johns Hopkins Bloomberg School of Public Health, Baltimore, MD USA; 2 Department of Pathology, Johns Hopkins School of Medicine, Baltimore, MD USA; 3 The James Buchanan Brady Urologic Institute and Department of Urology, Johns Hopkins School of Medicine, Baltimore, MD USA; 4 Sidney Kimmel Comprehensive Cancer Center at Johns Hopkins, Baltimore, MD USA

**Keywords:** miRNA, RNU6B, RNA stability, FFPE, Prostate cancer

## Abstract

**Background:**

The quantitative analysis of microRNA (miRNA) gene expression in archived formalin-fixed, paraffin embedded (FFPE) tissues has been instrumental to identifying their potential roles in cancer biology, diagnosis, and prognosis. However, it remains unclear whether miRNAs remain stable in FFPE tissues stored for long periods of time.

**Methods:**

Here we report Taqman real-time RT-PCR quantification of miR-21, miR-141, miR-221, and RNU6B small nuclear RNA (snRNA) levels from 92 radical prostatectomy specimens stored for 12–20 years in FFPE blocks. The relative stability of each transcript over time was assessed using general linear models. The correlation between transcript quantities, sample age, and RNA integrity number (RIN) were determined utilizing Spearman rank correlation.

**Results:**

All transcript levels linearly decreased with sample age, demonstrating a clear loss of miRNA stability and RNU6B snRNA stability over time. The most rapid rates of degradation were observed for RNU6B and miR-21, while miR-141 and miR-221 were more stable. RNA quality was not correlated with sample age or with miR-21, miR-221, or RNU6B snRNA levels. Conversely, miR-141 levels increased with RNA quality.

**Conclusions:**

MiRNA and snRNA levels gradually decreased over an eight year period in FFPE tissue blocks. Sample age was the most consistent feature associated with miRNA stability. The reference snRNA, RUN6B, was more rapidly degraded when compared to miR-141 and miR-221 miRNAs. Various miRNAs demonstrated differential rates of degradation. Quantitative miRNA studies from long-term archived FFPE tissues may therefore benefit from epidemiologic study design or statistical analysis methods that take into account differential storage-dependent transcript degradation.

**Electronic supplementary material:**

The online version of this article (doi:10.1186/s12885-016-3008-4) contains supplementary material, which is available to authorized users.

## Background

MicroRNAs are small non-coding transcripts that post-transcriptionally inhibit the translation and stability of specific mRNAs [1]. Deregulated miRNA expression has been reported in most every human cancer [2]. Moreover, numerous miRNAs are aberrantly expressed during disease development or progression, making miRNAs attractive as diagnostic or prognostic biomarkers [3]. Many of the miRNAs commonly deregulated in cancer have tumor suppressive or oncogenic properties when inhibited or over-expressed in cellular and animal models [4]. This collective study of miRNA gene expression in clinical tissues and miRNA function in cancer models has been crucial to our current understanding of the roles miRNAs may play in human cancer.

Archival formalin-fixed, paraffin-embedded (FFPE) tissues have been an invaluable resource for gene expression studies in cancer biology. These samples provide a historical record of tissue histology, protein and nucleic acid expression that can be correlated with long-term follow up of disease outcomes. Properly fixed and embedded samples can be stored indefinitely at room temperature without loss of structural integrity. However, processing and long-term storage can result in the damage and degradation of proteins and RNA transcripts. The stability of coding mRNA in FFPE specimens is dependent upon many factors, including processing methods, storage time and transcript abundance [5, 6]. RNA quality measurements, such as RNA integrity number (RIN) score, are currently applied to identify more reliable specimens for mRNA gene expression analyses [7]. It remains unclear, however, how well analyses of transcript size represent the quality or stability of small non-coding RNAs, such as miRNAs.

Comparative analyses of FFPE and rapidly frozen tissues suggests that miRNAs are unusually stable when compared to longer mRNA transcripts [8, 9]. In point of fact, the intentional degradation of RNA samples by exposure to high temperatures reduces RIN and mRNA stability without apparent influence on miRNA levels [10]. However, the literature has reached variable conclusions regarding miRNA stability in FFPE specimens stored for long periods of time [9–17]. There is therefore a need to further study miRNA stability in aged FFPE samples. Here we present new data on miRNA and RNU6B snRNA stability from 92 FFPE radical prostatectomy specimens that were processed at a single institution and stored for 12–20 years. The results indicate steady degradation of miRNAs over time and indicate that different miRNAs have different stabilities. The commonly applied reference snRNA transcript, RNU6B, showed differential stability from some miRNAs over time. These results signify a need to consider sample age and potential differences between miRNA and other snRNA stabilities in miRNA expression analyses from aged FFPE tissue samples.

## Methods

### Archival samples and RNA extractions

Total RNA from FFPE radical prostatectomy specimens was previously obtained from a cohort of 92 patients who underwent surgery at Johns Hopkins between 1993 and 2001, and were followed for recurrence until 2004 [18]. Briefly, malignant regions were identified by a pathologist and two 0.6 mm tumor cores were obtained from each sample. Samples were deparaffinized with xylene, extracted with ethanol, and protease digested. Total RNA was extracted with the RecoverAll Total Nucleic Acid Isolation Kit (Ambion, Grand Island, NY) and stored at −80 °C. RNA quantity was assessed by Nanodrop spectroscopy (Thermo Scientific, Wilmington, DE) and RNA integrity score were obtained with Agilent RNA Pico kit and the 2100 Bioanalyzer Instrument (Agilent Technologies, Wilmington, DE) as per manufacturer instructions.

### Quantitative RT-PCR

Reverse transcription and quantitative PCR were previously performed using the Taqman MicroRNA Reverse Transcription Kit and Applied Biosystems qRT-PCR probe and primer sets (Applied Biosystems, Grand Island, NY) [18]. Briefly, 2 ng of RNA from FFPE samples was applied to RT and QPCR was performed with the Bio-Rad MyiQ single color RT-PCR detection system (Bio-Rad Laboratories, Hercules, CA). Standard curves (2 ng/μl to 0.016 ng/μl) for RNU6B and for each miRNA transcript were generated from cell lines known to express each miRNA (PC3 for miR-221; LNCaP for miR-141; and MCF-7 for miR-21). The resulting standard curves and results were used to determine assay linearity, qRT-PCR efficiency, and SQmean.

### Statistical analyses

General linear models were utilized to compare RNA transcript stability rates in models with and without interaction. If the interaction term was deemed insignificant (parallel slopes) comparisons were further assessed utilizing analysis of covariance (ANCOVA). Statistical comparisons with RNU6B were applied using levels determined from each cell line standard curve (PC3 for miR-221; LNCaP for miR-141; and MCF-7 for miR-21). The correlation between transcript quantity and RIN or FFPE block age (sample age) were determined by Spearman rank correlation, and RNU6B levels derived from all cell line standard curves reached the same conclusions. For ease of reporting only RNU6B derived from PC3 standard curves are reported for the correlation analysis. Two-sided tests were conducted using R (version 3.2.3), and in all analyses, *p*-values <0.05 were considered to be statistically significant.

## Results

### Loss of miRNA and snRNA stability in archival FFPE specimens

**Fig. 1:**
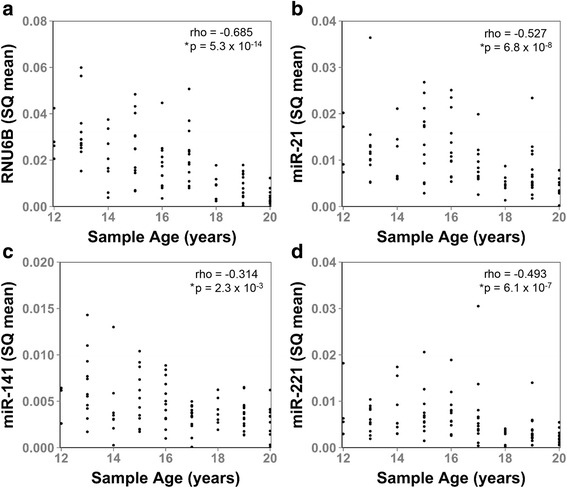
Loss of miRNA and RNU6B snRNA signal with sample block age. The association between small non-coding RNA transcript level (SQ mean) and sample age were analyzed for (**a**), RNU6B, (**b**), miR-21, (**c**), miR-141, and (**d**), miR-221 in radical prostatectomy specimens stored for 12–20 years in FFPE blocks. The levels of all four transcripts were inversely associated with sample block age by Spearman Rank Correlation. Spearman rank correlation coefficients (rho) and *p*-values are indicated for each sample. Asterisk indicates statistical significance

Over the last several decades the Johns Hopkins retropubic radical prostatectomy program has treated thousands of men with localized prostate cancer [19–21]. Archival specimens from these patients provide a unique resource to study the effects of long term FFPE tissue block storage on miRNA stability. As a single institution study of a single type of cancer, these samples underwent standardized processing, fixation, and storage procedures. Therefore, they present a unique and well-controlled resource for miRNA analyses. Tissue blocks from a previously defined nested-case control cohort of 92 radical prostatectomy patients, treated at Johns Hopkins Hospital between 1993 and 2001, were identified and total RNA was extracted from pathologist-defined tumor tissue [18]. The characteristics of patient and tumor samples are provided in Table 1. Transcript levels of miR-21, miR-141, miR-221, and the commonly applied reference gene, RNU6B snRNA, were quantified by Taqman quantitative RT-PCR assay. The results demonstrate a clear and statistically significant inverse association between transcript quantity and FFPE block age for all four small non-coding RNA transcripts by Spearman rank correlation analysis (Fig. 1, Additional file 1: Table S1). These results indicate a continuous loss of miRNA and RNU6B snRNA transcript stability over storage time.

**Table 1:** Characteristics of men who underwent prostatectomy for clinically localized disease, Johns Hopkins Hospital

*N*	92
Mean ± standard deviation age (years)	58.7 ± 6.8
White (%)	89.0
Positive surgical margins (%)	28.3
Median (interquartile range) pre-surgery PSA concentration (ng/mL)	8.0 (6.1, 12.4)
Pathologic Gleason sum (%)	
6	25.0
7	53.3
8+	21.7
Pathologic stage (%)	
T2	16.3
T3a	43.5
T3b/N1	40.2

### Small non-coding RNA stability and RNA Quality

**Fig. 2:**
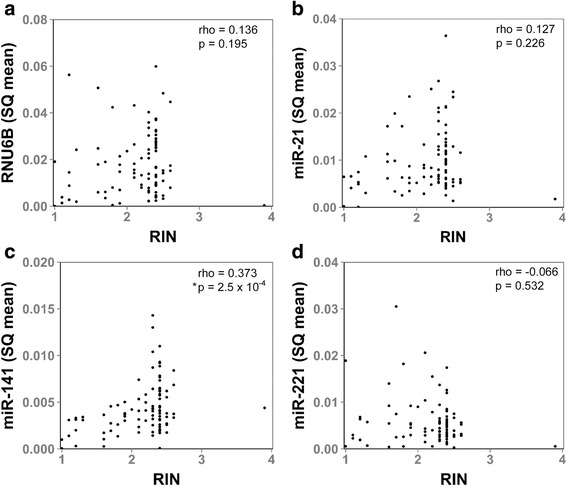
Evaluation of miRNA and RNU6B snRNA levels with RNA Integrity Number. The association between small non-coding RNA transcript level (SQ mean) and sample RNA Integrity Number (RIN) were analyzed for (**a**), RNU6B, (**b**), miR-21, (**c**), miR-141, and (**d**), miR-221 in RNA isolated from FFPE blocks. Sample RIN was associated with miR-141 levels, but not RNU6B snRNA, miR-21, or miR-221 levels. Spearman rank correlation coefficients (rho) and *p*-values are indicated for each sample. Asterisk indicates statistical significance

The quality of RNA from older FFPE samples is generally considered to be poor. To investigate whether RNA quality is indicative of miRNA and snRNA transcript levels, we calculated the RIN for each sample. RIN scores ranged from 1 to 3.9 (Average 2.1 ± 0.5). No association was found between sample RIN and FFPE block age (Additional file 1: Figure S1). Moreover, no statistically significant correlation was found between RIN and RNU6B snRNA, miR-21, or miR-221 transcript levels by Spearman rank correlation (Fig. 2a, b and d). However, a significant correlation was observed between RIN and miR-141 transcript levels (Fig. 2c), indicating that RNA quality can be associated with the stability and detectability of some miRNAs. Collectively, these results identify FFPE block age as the most consistent feature associated with miRNA and snRNA transcript detectability and stability. The results of these statistical analyses are summarized in Additional file 1: Table S2.

### Differential stability between small non-coding RNA transcripts

**Fig. 3:**
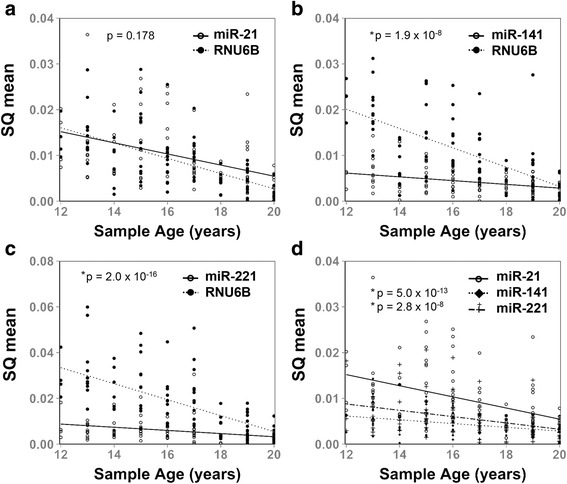
Differential relative stability of miRNAs and RNU6B snRNA in FFPE samples stored for long periods of time. Linear associations between miRNA SQ mean levels, or RNU6B SQ Mean levels, and FFPE Sample Age were analyzed and compared. **a** RNU6B and miR-21 had similar stabilities over 12–20 years (p-interaction = 0.194, *p* = 0.178 by analysis of covariance). **b** miR-141 was significantly more stable than RNU6B snRNA over 12–20 years (p-interaction = 1.9 × 10^−8^). **c** miR-221 was significantly more stable than RNU6B snRNA over 12–20 years (p-interaction = 2.0 × 10^−16^). **d** miR-221 was significantly more stable than miR-21 (*p* = 5.0 × 10^−14^) and miR-141 was significantly more stable than miR-21 (*p* = 2.8 × 10^−8^) over 12–20 years

Mature miRNAs are approximately 22 nucleotides in length while common endogenous reference controls, such as RNU6B snRNA, are slightly longer (Table 2). Given the differences in length and function between miRNAs and reference snRNAs, we investigated whether there may be corresponding differences in signal stability over time by general linear models. RNU6B and miR-21 were found to have comparable stabilities over time (Fig. 3a). However, the rate of RNU6B snRNA degradation was significantly more rapid than that of miR-141 and miR-221 (Fig. 3b–c). Accordingly, miR-21 was also found to have a more rapid degradation rate when compared to miR-141 and miR-221 (Fig. 3d). These data support that commonly studied microRNAs and reference controls can have significantly different stabilities in FFPE tissues stored over long periods of time. These results may reflect differences in small non-coding RNA sequence, function, or association with proteins. They further suggested that quantitative miRNA studies from long-term archived FFPE tissues may benefit from epidemiologic study design or statistical analysis methods that take into account differential storage-dependent transcript degradation.

**Table 2:** Characteristics of miRNA and snRNA Transcripts

Transcript	Sequence	Length (nt)	GC Content
hsa-miR-141	UAACACUGUCUGGUAAAGAUGG	22	40.90%
hsa-miR-21	UAGCUUAUCAGACUGAUGUUGA	22	36.40%
hsa-miR-221	AGCUACAUUGUCUGCUGGGUUUC	23	47.80%
RNU6B	CTGCGCAAGGATGACACGCAAATTCGTGAAGCGTTCCATATTTTT	45	44.40%

## Discussion

The first reported miRNA gene, lin-4, was described in *C. elegans* in 1993 [22]; the same year that the first surgical samples in this study were isolated. In a perfect world these samples would remain unchanged over time; however, it is well known that processing and storage can lead to significant RNA degradation [5, 6]. The influence of such parameters on miRNA detection and stability remains debatable.

A recent study of six miRNAs in colorectal tissue blocks stored for up to 28 years found no significant effects of sample block age on miRNA detection [12]. Similar observations were reported for miR-181b and 5S ribosomal RNA from blocks as old as ten years [8]. Deep sequencing analyses of miRNAs from multiple different types of tissue, stored for 2 to 9 years, have also found no significant change in miRNA detection with sample age [9]. In contrast, others have reported significant miRNA loss with extended FFPE block storage times. Comparison of miRNA expression from 1 to 11 year old FFPE samples reported a clear and gradual loss of miRNA signal with storage time [16]. Gradual loss of miRNA signal was also reported from FFPE samples of tongue carcinoma [14], with detectable signal loss after only one year of storage. In yet another study, miRNA levels from human tissues processed and stored as FFPE blocks for more than 10–20 years showed a nearly 50% decrease in miRNA accessibility [15]. These few examples of conflicting results underscore the need to better understand if and how FFPE block storage time may influence miRNA detection.

Here we report the effect of FFPE block storage time on the stability of three frequently studied miRNAs, and one of the most commonly utilized miRNA reference genes, in prostate cancer specimens stored for up to 20 years. Importantly, this study was performed on samples of a single tissue type that were isolated and processed by a single institution. Therefore tissue type, processing methodology and storage have been consistent. Our results support that miRNAs and snRNAs are not stable over time in FFPE samples stored for over 12 years (Fig. 1).

There are several factors that can contribute to the loss of RNA stability in FFPE blocks including fixation time, fixation method, or exposure to oxidation, extreme temperatures, or light [10, 13, 17]. Capillary electrophoresis analyses and RIN score are standard methods for evaluating RNA quality from such clinical specimens. These methods primarily focus on nucleotide fragment size distribution; therefore, they may not distinguish small RNA transcripts from mRNA degradation products. In our study, RIN score was not associated with small non-coding RNA stability in three of the four transcripts studied (Fig. 2). Moreover, two identically sized miRNAs, miR-21 and miR-141, had significantly different stabilities in this sample set (Fig. 3). On the other hand, miR-141 levels were found to be associated with RIN score. These results reflect a common need to identify better tools for assessing miRNA quality in clinical specimens. It is notable that our sample set had a limited range of RIN scores and did not include any samples younger than 12 years of age. Consequently, a more clear correlation between miRNA stability, sample age, and RIN score may have been observed with a broader range of sample ages and qualities.

One interesting observation from this study is the apparent differential stability between some miRNAs. MiR-221 and miR-141 were significantly more stable over time when compared to miR-21 and RNU6B (Fig. 3). This supports previous reports of differential stability between different miRNAs [9]. Perhaps differences in miRNA function, localization, or extent and stability of binding to other macromolecules help account for these observations. Further studies are needed to characterize these features and to identify mechanisms that contribute to or inhibit miRNA degradation over time.

## Conclusions

We report a linear loss of miRNA and RNU6B snRNA signal with sample age in FFPE blocks stored for 12–20 years. Some miRNAs were more stable than others. FFPE block age was the most consistent feature associated with miRNA and snRNA stability. It is important to emphasize the poor stability of RNU6B snRNA, when compared to some miRNAs, because it is one of the most widely utilized reference genes for miRNA expression normalization. Our results suggest that it would be beneficial to consider sample block age, rather than RNA quality, in miRNA expression analyses from older FFPE samples. For example, we have previously noted trends in miRNA and RNU6 snRNA stability and have adjusted analyses by calendar year of prostatectomy [18]. Similar adjustments have been made in other studies [14]. Future research is needed to identify alternative methods for assessing miRNA and snRNA quality in older FFPE samples, and for miRNA expression comparisons and normalization.

## Additional file

Additional file 1: Table S1. Spearman’s rank correlation of sample value and FFPE block age. **Table S2.** Spearman’s rank correlation of sample value and RNA Integrity Number. **Figure S1.** Association of RIN and Sample Block Age. No association was observed between sample age and RNA Integrity Number (RIN). (DOCX 258 kb)

## Abbreviations

ANCOVA: Analysis of covariance
FFPE: Formalin-fixed, paraffin embedded
miRNA: MicroRNA
mRNA: Messenger RNA
RIN: RNA integrity number
