# Supplementary material for: Differential long-term stability of microRNAs and RNU6B snRNA in 12–20 year old archived formalin-fixed paraffin-embedded specimens
Source: BMC Cancer. 2017 Jan 6;17:32. doi: 10.1186/s12885-016-3008-4 (PMC5219687; doi:10.1186/s12885-016-3008-4)
Supplement: Additional file 1: Table S1. — Spearman’s rank correlation of sample value and FFPE block age. Table S2. Spearman’s rank correlation of sample value and RNA Integrity Number. Figure S1. Association of RIN and Sample Block Age. No association was observed between sample age and RNA Integrity Number (RIN). (DOCX 258 kb) [file 12885_2016_3008_MOESM1_ESM.docx]

**Supplementary Information**

Supplementary Tables:

| **Supplementary Table S1: Spearman's rank correlation of sample value and FFPE block age** | | | |
| --- | --- | --- | --- |
|  | N | rho | p-value |
| PC3 RNU6B | 92 | -0.685 | <.001 |
| miR-21 | 92 | -0.527 | <.001 |
| miR-141 | 92 | -0.314 | 0.002 |
| miR-221 | 92 | -0.493 | <.001 |

| **Supplementary Table S2: Spearman's rank correlation of sample value and RNA Integrity Number** | | | |
| --- | --- | --- | --- |
|  | N | rho | p-value |
| PC3 RNU6B | 92 | 0.136 | 0.2 |
| miR-21 | 92 | 0.127 | 0.2 |
| miR-141 | 92 | 0.373 | <.001 |
| miR-221 | 92 | -0.066 | 0.5 |

Supplementary Figures:


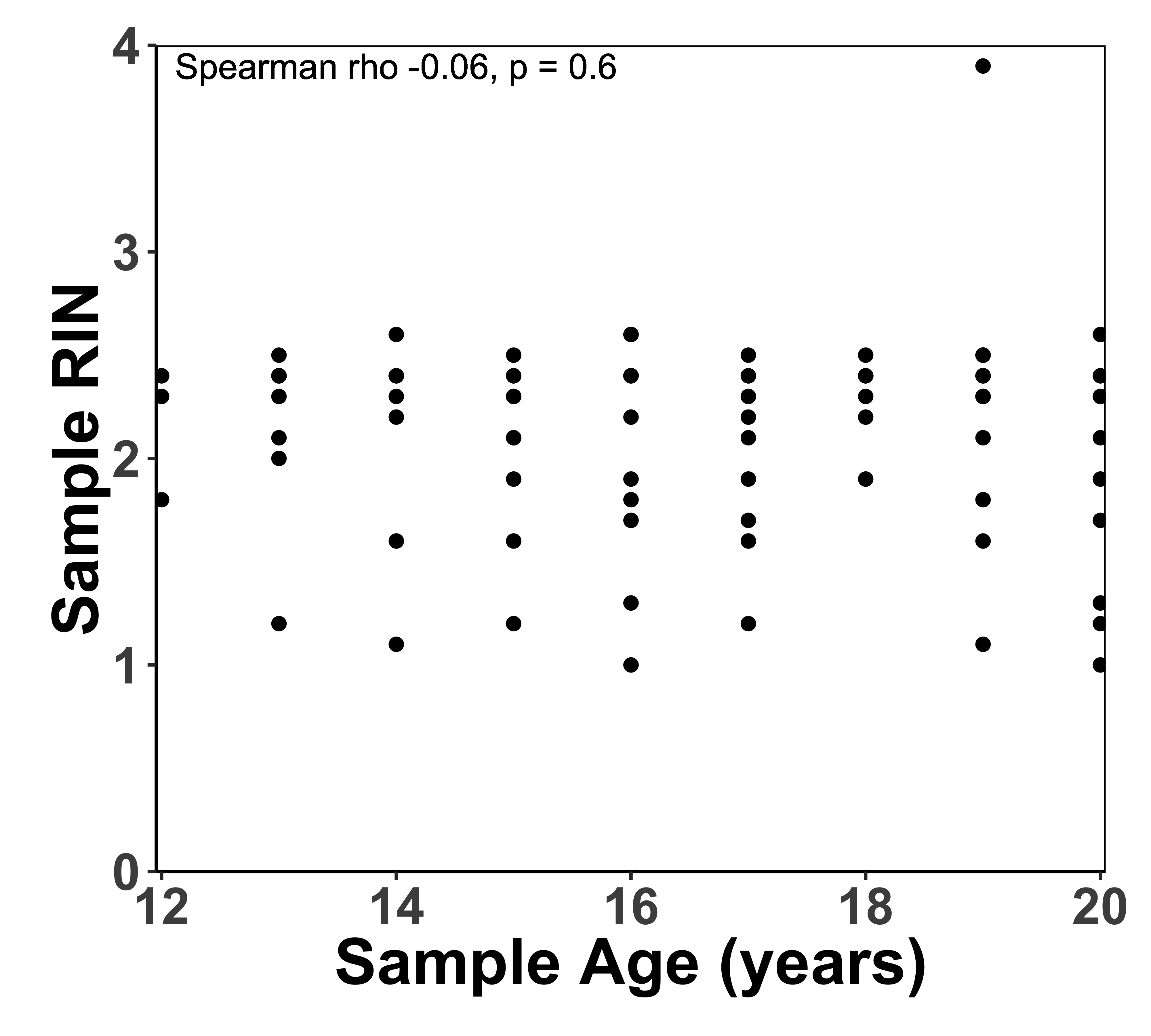


**Supplementary Figure 1. Association of RIN and Sample Block Age.** No association was observed between sample age and RNA Integrity Number (RIN).
